# New aspects of grassland recovery in old‐fields revealed by trait‐based analyses of perennial‐crop‐mediated succession

**DOI:** 10.1002/ece3.2869

**Published:** 2017-03-12

**Authors:** András Kelemen, Béla Tóthmérész, Orsolya Valkó, Tamás Miglécz, Balázs Deák, Péter Török

**Affiliations:** ^1^ MTA's Post Doctoral Research ProgramMTA TKIBudapestHungary; ^2^ Department of EcologyUniversity of DebrecenDebrecenHungary; ^3^ MTA‐DE Biodiversity and Ecosystem Services Research GroupDebrecenHungary

**Keywords:** alfalfa, C‐S‐R strategies, functional diversity, leaf traits, resource acquisition, seed traits

## Abstract

Classical old‐field succession studies focused on vegetation changes after the abandonment of annual croplands or on succession after the elimination of cultivated crops. Perennial‐crop‐mediated succession, where fields are initially covered by perennial crops, reveals alternative aspects of old‐field succession theories. We tested the validity of classical theories of old‐field succession for perennial‐crop‐mediated succession. We formulated the following hypotheses: (1) functional diversity increases with increasing field age; (2) resource acquisition versus conservation trade‐off shifts toward conservation at community level during the succession; (3) the importance of spatial and temporal seed dispersal decreases during the succession; and (4) competitiveness and stress‐tolerance increases and ruderality decreases at community level during the succession. We studied functional diversity, trait distributions and plant strategies in differently aged old‐fields using chronosequence method. We found increasing functional richness and functional divergence, but also unchanged or decreasing functional evenness. We detected a shift from resource acquisition to resource conservation strategy of communities during the succession. The role of spatial and temporal seed dispersal was found to be important not only at the initial but also at latter successional stages. We found an increasing stress‐tolerance and a decreasing ruderality during succession, while the competitiveness remained unchanged at the community level. Despite the markedly different starting conditions, we found that classical and perennial‐crop‐mediated old‐field successions have some similarities regarding the changes of functional diversity, resource acquisition versus conservation trade‐off, and seed dispersal strategies. However, we revealed also the subsequent differences. The competitive character of communities remained stable during the succession; hence, the initial stages of perennial‐crop‐mediated succession can be similar to the middle stages of classical old‐field succession. Moreover, the occupied functional niche space and differentiation were larger in the older stages, but resources were not effectively utilized within this space, suggesting that the stabilization of the vegetation requires more time.

## 1 Introduction

Studying old‐field succession has a long tradition in ecology; Rejmánek and Katwyk (2004) enumerated 1,511 studies for the 1901–1990 period dealing with various aspects of old‐field succession. In spite of this, most studies concentrated on succession after the abandonment of annual croplands or on vegetation changes after the complete removal of former cultivated crop (see Rejmánek & Katwyk, 2004; we call this type of succession “classical old‐field succession” in the forthcomings). To “perennial‐crop‐mediated succession,” that is, succession in fields initially covered by perennial crops (without its removal) received less attention (but see Li, Xu, & Wang, 2008; She, Shao, Timm, & Reichardt, 2009; Török et al., 2011).

This lack of information can be considered an important gap of knowledge which should be treated. This is even more important as the perennial crops are widely grown with a total area of approximately 164 million hectares worldwide (FAOSTAT, 2012). Alfalfa (*Medicago sativa*) has one of the largest cultivated area out of the perennial forage legumes with approximately 30 million ha worldwide (Yuegao & Cash, 2009). Abandonment of croplands is still increasing worldwide, and large areas of perennial croplands—especially alfalfa fields—are also increasingly subjected to abandonment (Cramer, Hobbs, & Standish, 2008; Török et al., 2011). From the conservation viewpoint, the perennial croplands—mainly the nitrogen‐fixing legume fields—can be promising starting stages of grassland recovery (Li et al., 2008; She et al., 2009; Török et al., 2011; Valkó et al., 2016). All these facts underline the necessity of understanding old‐field succession and vegetation dynamics in case of perennial‐crop‐mediated succession.

The scientific methods in succession studies have been changed over time, recently the trait‐based functional diversity came into main focus instead of the former species identity‐based approaches (Navas, Roumet, Bellmann, Laurent, & Garnier, 2010; Raevel, Violle, & Munoz, 2012). In most cases the analysis of functional diversity provides a more straightforward measure of ecosystem processes than the species diversity, because functional diversity is directly linked to ecosystem functioning (Garnier & Navas, 2012). Moreover, a trait‐based functional approach gives an opportunity for broad generalization of the findings allowing the comparison between communities with distinct species pools (Lepš, de Bello, Šmilauer, & Doležal, 2011; Westoby, 1998). While successional processes are often unpredictable at the level of species composition, functional changes are rather deterministic (Prach & Walker, 2011; Purschke et al., 2013). Functional trait‐based approaches are appropriate to reveal the mechanisms shaping the niche utilization and resource acquisition/conservation trade‐off (de Bello et al., 2009; Botta‐Dukát & Czúcz, 2016; Garnier & Navas, 2012). The trait‐based functional changes during the succession can be described by multitrait functional diversity indices (functional richness, evenness, and divergence (FRic, FEve, and FDiv, respectively); Villéger, Mason, & Mouillot, 2008) and trait‐based plant strategies (i.e., C‐S‐R strategies; Grime, 1979; Hunt et al., 2004; Pierce, Brusa, Vagge, & Cerabolini, 2013).

Former papers, focusing on the perennial‐crop‐mediated succession, studied the changes in species diversity, growth form spectrum, and biomass fractions during succession (Li et al., 2008; She et al., 2009; Török et al., 2011). Studies using multitrait methods focused on classical old‐field succession, which means the succession of annual croplands or the succession after the removal of former cultivated crop (see Garnier & Navas, 2012). These studies revealed the general processes of old‐field succession.

Functional diversity was found to be increasing during succession indicating a higher rate of filled niche space, more complete resource use, and niche differentiation (Garnier & Navas, 2012; Purschke et al., 2013) in sense of the niche complementarity hypothesis (Tilman, 1997). Species with rapid resource acquisition ability are gradually replaced by others characterized by efficient conservation of resources (Kazakou et al., 2007; Navas et al., 2010; Vile, Shipley, & Garnier, 2006). These changes are well reflected at the level of individual leaf traits, such as the decrease in specific leaf area (SLA) and the increase in leaf dry matter content (LDMC) typically found in classical old‐field succession (Garnier et al., 2004; Kazakou, Vile, Shipley, Gallet, & Garnier, 2006; Navas et al., 2010; Purschke et al., 2013).

Functional traits related to seed dispersal are also important drivers of vegetation processes (Gross & Emery, 2007); succession theories predict the higher importance of effective seed dispersal ability (e.g., anemochory, epizoochory) in initial stages of old‐field succession compared with the latter ones (Dölle, Bernhardt‐Römermann, Parth, & Schmidt, 2008; Purschke et al., 2013). Besides the seed rain, species established from their persistent seed banks can also reach high abundances in early communities of secondary succession (Kiss, Valkó, Tóthmérész, & Török, 2016).

C‐S‐R strategy theory is a holistic approach of classification of plants based on their responses to principal types of environmental factors (stress and disturbance) and to competition of other plants (Grime, 1979; Hodgson, Wilson, Hunt, Grime, & Thompson, 1999; Pierce et al., 2013). Former researches found the gradual replacement of ruderal species by competitors and stress‐tolerators in course of old‐field succession (Dölle et al., 2008; Navas et al., 2010; Prévosto et al., 2011).

Our aim was to test the validity of the above‐described theories of classical old‐field succession for perennial‐crop‐mediated succession. Based on these classical theories we formulated the following hypotheses: (1) functional diversity increases with increasing field age; (2) the resource acquisition versus conservation trade‐off shifts toward the conservation at community level during the succession; (3) the importance of both spatial and temporal seed dispersal decreases during the succession; and (4) competitiveness and stress‐tolerance increases and ruderality decreases at community level during the succession.

## 2 Materials and Methods

### 2.1 Study area and sampling

Our study sites are located in the Hortobágy region (Eastern Hungary), which is a part of the Great Hungarian Plain. The climate of this region is continental with 550 mm mean annual precipitation and 9.5°C mean annual temperature (Lukács et al., 2015). We applied the chronosequence method (Walker, Wardle, Bardgett, & Clarckson, 2010) and studied altogether twelve extensively managed alfalfa fields (1‐, 3‐, 5‐ and 10‐year‐old) with three spatial replicates per age group. The fields were situated on loess plateaux characterized by elevations from 87 to 94 m a.s.l. (Kelemen et al., 2014; Török et al., 2011), within a 50‐km radius (GPS coordinates for the center: N47°26′; E21°01′). None of the study sites were directly connected to loess grasslands (which was the original vegetation type of our study sites; Deák et al., 2014; Tóth & Hüse, 2014); however, there were loess grasslands in their close proximity (Török et al., 2011; Valkó et al., 2016). The alfalfa fields were extensively managed which means that they were mown twice a year but there were no further management. In the study region, alfalfa is not removed during the abandonment of fields. Therefore, it was not possible to establish local control sites characterized by complete removal of alfalfa after abandonment. The vegetation of young alfalfa fields is generally dominated by alfalfa which is replaced by perennial grasses afterward, because alfalfa is a short‐lived perennial crop (Török et al., 2011). This replacement is the most pronounced between the third and fifth years; therefore, the fields older than 5 years are characterized by perennial grass dominance (Török et al., 2011). In each of the twelve sites (four age categories, three sites per age category) three 5 m × 5 m blocks were randomly designated. Within each block, the percentage covers of vascular plants were recorded in four 1 m × 1 m plots (12 plots per site, 36 plots per age group, altogether 144 plots) in early June 2009, before the first mowing.

### 2.2 Acquisition of plant trait data

We used the following leaf traits as continuous traits to calculate functional diversity indices: specific leaf area (SLA; mm^2^/mg), leaf dry matter content (LDMC; mg/g), leaf area (LA; mm^2^). Leaf trait data were obtained from the LEDA database (Kleyer et al., 2008). We used further traits to evaluate the roles of spatial (terminal velocity and epizoochory ranking index) and temporal seed dispersal (seed bank type; Thompson, Bakker, & Bekker, 1997) in vegetation dynamics. Terminal velocity (m/s) and epizoochory ranking index (ranging from 0 to 1, where 0 indicates species with the lowest and 1 indicates species with the highest potential for epizoochory) were derived from D^3^ database (Hintze et al., 2013). We used seed bank type as categorical trait with three categories: (1) transient (seeds persist in the soil for less than 1 year); (2) short‐term persistent (seeds persist in the soil for at least 1 year, but less than 5 years); and (3) long‐term persistent (seeds persist in the soil for at least 5 years) following the classification of Thompson et al. (1997). In case of several species, Thompson et al. (1997) gives more than one seed bank type categories; in these certain cases we took the most frequent type into consideration.

It was also necessary to obtain data about canopy height, flowering period, flowering start, leaf dry weight (LDW) and lateral spread of each species for the trait‐based C‐S‐R classification based on Hodgson et al. (1999). Flowering period (months) and flowering start (categorical) data were obtained from Király (2009), while leaf dry weight (mg) data originated from the LEDA database (Kleyer et al., 2008). To define the lateral spread categories we used the methods of Hodgson et al. (1999) based on information derived from CLO‐PLA database (Klimešová & de Bello, 2009).

### 2.3 Data analysis

We used three multitrait functional diversity (FD) indices proposed by Villéger et al. (2008) to describe community‐level functional changes during succession. These three complementary indices (functional richness—FRic, functional evenness—FEve, and functional divergence—FDiv) are statistically independent from each other and are suitable to evaluate the underlying mechanisms of vegetation changes (Mason, Mouillot, Lee, & Wilson, 2005; Villéger et al., 2008). FRic describes the volume of filled functional space; FEve measures both the regularity of species distribution within the functional space and the evenness of abundance across species; and FDiv represents how abundance is distributed within the occupied functional space (Mason et al., 2005; Villéger et al., 2008). The calculation of these FD indices was based on leaf traits (SLA, LDMC, LA) and performed in R programme using FD library (Laliberté & Legendre, 2010).

We also calculated community weighted means (CWM) for these leaf traits and for the two traits related to spatial seed dispersal (terminal velocity and epizoochory ranking index). In CWM calculations we used the relative species covers for weights. In case of the only one studied categorical trait (seed bank type), we gave the relative cover of the categories in each plot.

We used a complex scheme of plant strategies, the C‐S‐R functional types proposed by Grime (1979). We used the method of Hodgson et al. (1999) based on functional traits to place species in the C‐S‐R space. This classification method uses six predictor variables (canopy height, LDMC, flowering period, lateral spread, LDW, SLA) in case of graminoids and seven predictor variables (the above‐mentioned six traits and flowering start) in case of forbs. Species were classified to C‐S‐R categories with a freely available Excel macro (http://people.exeter.ac.uk/rh203/allocating_csr.html). As a result of the classification the species were assigned into one of the 19 mixed C‐S‐R categories (Hodgson et al., 1999). After that, we calculated the community‐level C‐S‐R distribution (C‐S‐R signature) of each sample using the method of Hunt et al. (2004) with another freely available Excel macro (http://people.exeter.ac.uk/rh203/csr_signature.html). This community‐level approach provides the proportion of C, S, and R characters (C, S and R coordinates) for each sample (Hunt et al., 2004).

We used linear mixed‐effect models (LMEs) with nested design for exploring the vegetation changes during the succession. In LMEs we set field age as fix factor and site as random factor nested in field age. Dependent variables were the multitrait FD indices, CWMs of single traits, relative covers of seed bank type categories and proportion of C, S, and R characters. To compare the values of dependent variables in the differently aged alfalfa fields we used Tukey's test (*p *<* *.05). LMEs were calculated using STATISTICA 10.0 (StatSoft Inc., Tulsa, OK, USA). During the computations we performed most of the analyses (except for analyses related to seed dispersal) in two ways: (1) we analyzed the vegetation changes based on the whole community including also the cover of alfalfa; and (2) we excluded the cover of alfalfa from calculations and concentrated on the functional characters of colonizers. In case of traits related to seed dispersal we focused on vegetation trends analyzed without counting alfalfa, because we were interested in the spatial and temporal seed dispersal of species spontaneously colonizing alfalfa fields.

## 3 Results

### 3.1 Functional diversity indices and leaf traits

FRic increased with increasing field age both in case of the calculations with and without alfalfa, and the age effects were marginally significant (Table 1). No field age effect was detected on FEve when the alfalfa was included, but there were significantly negative age effects when the alfalfa was excluded from calculation (Table 1). In case of FDiv we detected a positive age effect, which was marginally significant in the calculation including alfalfa and was significant when alfalfa was excluded from the analysis (Table 1).

**Table 1:** Age effects in case of multitrait diversity indices based on the results of LME analyses

	Age	Trend	*F*	1‐year‐old	3‐year‐old	5‐year‐old	10‐year‐old
*With alfalfa*							
FRic	#	↑	3.025	0.139 ± 0.011^AB^	0.113 ± 0.012^A^	0.199 ± 0.011^C^	0.159 ± 0.010^B^
FEve	n.s.	—	0.611	0.576 ± 0.018	0.540 ± 0.033	0.521 ± 0.019	0.518 ± 0.020
FDiv	#	↑	3.728	0.587 ± 0.034^A^	0.642 ± 0.038^A^	0.663 ± 0.024^A^	0.817 ± 0.018^B^
*Without alfalfa*							
FRic	#	↑	3.100	0.135 ± 0.012^AB^	0.114 ± 0.012^A^	0.198 ± 0.011^C^	0.159 ± 0.010^B^
FEve	*	↓	5.198	0.744 ± 0.018^B^	0.695 ± 0.026^B^	0.564 ± 0.019^A^	0.529 ± 0.021^A^
FDiv	**	↑	10.959	0.745 ± 0.02^A^	0.707 ± 0.017^A^	0.852 ± 0.016^B^	0.838 ± 0.017^B^

Trends denote the direction of changes during the succession. The means and SEs of indices were given in the table. The different superscripted letters indicate significant differences obtained with Tukey's test (*p *<* *.05), letter “A” signs the lowest value in every case. Notations: n.s.: nonsignificant; #: marginally significant (.05 < *p *<* *.1); *.01 < *p *≤* *.05; **.001 < *p *≤* *.01; —: no obvious trend; ↑: increasing during the succession; ↓: decreasing during the succession.

**Figure 1:**
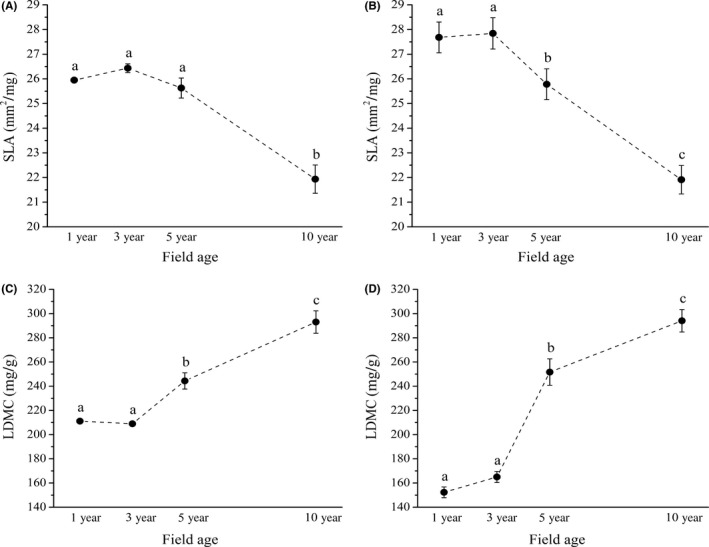
Community weighted means of SLA and LDMC during the succession (CWM ± SE). Subfigures: CWMs of SLA calculated with alfalfa (A); CWMs of SLA calculated without alfalfa (B); CWMs of LDMC calculated with alfalfa (C); CWMs of LDMC calculated without alfalfa (D). Different letters denote significant differences obtained with Tukey's test (*p *<* *.05)

CWM of SLA was negatively affected by field age in both cases; however, the age effects were only marginally significant (with alfalfa: *F* = 3.86; *p *=* *.056; without alfalfa: *F* = 2.60; *p *=* *.098; Figure 1A, B). Age effect was significantly positive in case of CWM of LDMC in both types of analyses (with alfalfa: *F* = 6.60; *p *=* *.014; without alfalfa: *F* = 10.05; *p *=* *.004; Figure 1C, D). We did not detect significant age effect in case of CWM of LA (with alfalfa: *F* = 0.26; *p *=* *.854; without alfalfa: *F* = 0.80; *p *=* *.528).

### 3.2 Spatial dispersal and seed bank

**Figure 2:**
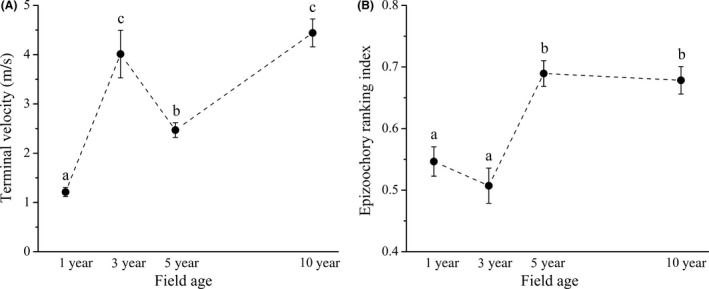
Community weighted means of terminal velocity (A) and epizoochory ranking index (B) during the succession (CWM ± SE). Different letters denote significant differences obtained with Tukey's test (*p *<* *.05)

**Figure 3:**
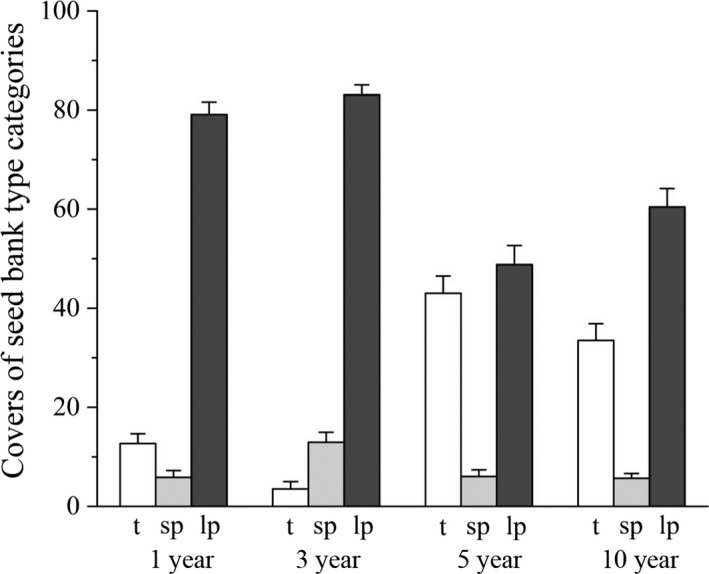
Relative covers of species with transient (t), short‐term persistent (sp), and long‐term persistent (lp) seed bank types in differently aged alfalfa fields (%+SE)

The field age positively affected the CWM of terminal velocity; however, the CWM was the highest in 3‐year‐old fields (*F* = 4.72; *p *=* *.035; Figure 2A). Age effect was significantly positive in case of CWM of epizoochory ranking index (*F* = 4.05; *p *=* *.049; Figure 2B). Cover of species group characterized by transient seed banks increased (*F* = 38.17; *p *<* *.001; Figure 3), while the cover of species group with long‐term persistent seed banks decreased with increasing field age (*F* = 14.32; *p *<* *.001; Figure 3). The age effect was not significant in case of species group characterized by short‐term persistent seeds (*F* = 1.14; *p *=* *.390; Figure 3).

### 3.3 Plant strategies

**Figure 4:**
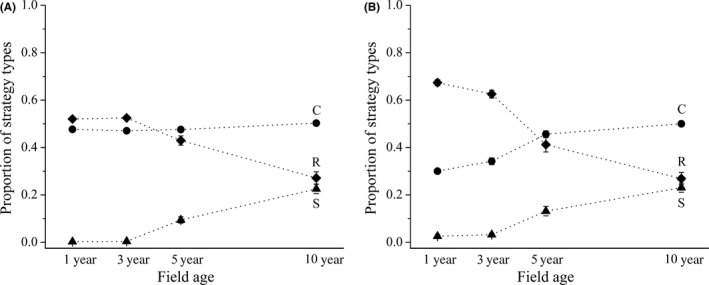
Proportions of C, S, and R strategy types (proportion ± SE) during the succession calculating with (A) or without alfalfa (B)

The values of C coordinates of communities did not change with field age when the alfalfa was included in calculation (*F* = 0.42; *p *=* *.742; Figure 4A); however, when it was excluded, the values significantly increased with increasing field age (*F* = 8.89; *p *=* *.006; Figure 4B). Values of S coordinates increased (with alfalfa: *F* = 11.38; *p *=* *.003; without alfalfa: *F* = 6.36; *p *=* *.016; Figure 4A, B), while the values of R coordinates decreased with increasing field age (with alfalfa: *F* = 5.29; *p *=* *.027; without alfalfa: *F* = 8.00; *p *=* *.009; Figure 4A, B), both with and without alfalfa.

## 4 Discussion

### 4.1 Functional diversity

In accordance with our first hypothesis two functional diversity indices (FRic, FDiv) were increased during the succession. In contrast, the unchanged (calculated with alfalfa) or decreased (without alfalfa) FEve did not support our expectations.

The increase in FRic in general means an increasing volume of filled functional niche space (Mason et al., 2005; Villéger et al., 2008). It indicates that in young alfalfa fields the potentially available resources were rather unused by community than in older fields. There is inherent positive relationship between FRic and species richness, because it is probable that more, functionally not identical species use higher volume of functional niche than fewer species (Villéger et al., 2008). In our study sites the species richness increased with increasing field age (see details in Török et al., 2011); thus, one of the reasons for the increasing FRic can be the increasing species richness. The young alfalfa fields were characterized by one dominant species, *Medicago sativa* with a certain set of used resources. Due to the high competitive ability of alfalfa there were only a few other species which probably used different resources (Garnier & Navas, 2012) occurred in young alfalfa fields. This phenomenon can result in a low volume of filled niche space. In young alfalfa fields the subordinate species group consisted of functionally similar species; therefore, they used only a low volume of the potentially available niches (Schumacher & Roscher, 2009). Afterward, the alfalfa was replaced by functionally more distinct species enlarging the volume of filled niche space (Purschke et al., 2013; Török et al., 2011).

FEve index measures both the regularity of species distribution in functional space and evenness of abundance across species referring to the degree of resource utilization within the occupied niche space (Mason et al., 2005; Villéger et al., 2008). FEve remained stable during the succession when we included the alfalfa in the analyses. This result indicated that the resource utilization within the occupied functional niche space remained unchanged. Without alfalfa, we detected a decrease in FEve, which means that the abundances and functional distances among species became less even during the succession (Villéger et al., 2008). Uneven distributions of traits and species abundances indicate that the occupied niche space was not effectively utilized (Mason et al., 2005).

FDiv represents how abundance is distributed within the occupied functional trait space indicating the rate of niche differentiation (Mason et al., 2005; Villéger et al., 2008). The increased FDiv indicated an increasing niche differentiation during the succession (Maire et al., 2012).

The detected trends in multitrait functional diversity measures showed that the volume of occupied functional niche space increased during the succession (increasing FRich). However, the resources in this extended niche space were not effectively utilized (constant or decreased FEve) in spite of the increasing niche differentiation (increasing FDiv). These results indicated the low stability of vegetation composition even in the 10‐year‐old fields. Thus, further changes in abundances and in species pool are expected (Fonseca & Ganade, 2001). In spite of the promising vegetation development toward the natural‐like grasslands (Li et al., 2008; Török et al., 2011), we suppose that the regeneration of natural‐like species abundances and biotic interactions requires more time than the 10‐year span of the study.

### 4.2 Resource use strategies

The community weighted means of SLA decreased with increasing field age and in parallel the CWMs of LDMC increased. These results confirmed our second hypothesis. Species with high SLA are characterized by fast growth and shade tolerance because of their efficient photosynthesis (Valladares & Niinemets, 2008; Westoby, 1998). The occurrence of species with high SLA could be accelerated by the N‐fixation of alfalfa (Garnier & Navas, 2012; Garnier et al., 2004; Kazakou et al., 2006). They could temporary overgrow the alfalfa after mowing and obtain more light which can be important in their survival in young fields with high alfalfa canopy. The results showed a pronounced shift from effective resource acquisition to effective resource conservation strategy at community level during the succession (Navas et al., 2010; Vile et al., 2006).

### 4.3 Seed dispersal

We hypothesized that the importance of both spatial dispersal (Dölle et al., 2008; Latzel et al., 2011) and seed bank decreases with time (Purschke et al., 2013). Our results only partly confirmed this hypothesis. In accordance with the hypothesis we found that the cover of species with effective wind‐dispersal potential (low terminal velocity) decreased. In contrast to our hypothesis the importance of epizoochorous dispersal increased during the succession. Although we detected high terminal velocity in the 3‐year‐old alfalfa fields, this was caused by the local invasion of a single species (*Convolvulus arvensis*) bearing seeds with high terminal velocity in one study site.

We found that in accordance with our third hypothesis, the cover of species with transient seed bank increased, while that of species with long‐term persistent seed bank decreased during the succession. These results are well in accordance with the findings of Purschke et al. (2013). However, the abundance of species with long‐term persistent seeds was the highest out of the three categories of Thompson et al. (1997) in each age group of alfalfa fields. These results indicated the vital role of local seed bank in the establishment of species in young alfalfa fields and also highlighted the importance of “founder effect” in succession of perennial crop fields (Albert et al., 2014; Grime, 1998). The new colonizers could also have a high importance, because the abundance of anemochorous species was high in young alfalfa fields, and there were also some species with high potential for epizoochorous dispersal. The cover of transient‐seeded species increased with field age. This latter result also indicated that not only the local seed bank determines the vegetation development during succession, but also the new colonizers are important. 5‐ and 10‐year‐old fields were characterized by higher abundance of species with high epizoochory ranking index, indicating the possibly important role of grazing wild animals, such as European roe deer (*Capreolus capreolus*) and European hare (*Lepus europaeus*) in vegetation processes. The alfalfa is a high‐quality forage; thus, can attract seed dispersing grazing animals (Selting & Irby, 1997; Will & Tackenberg, 2008). The 5‐ and 10‐year‐old fields were dominated by perennial grasses (e.g., *Alopecurus pratensis*,* Elymus repens*,* Poa angustifolia*), which are easily dispersed by mammals via epizoochory because of their caryopses with appendages (Hintze et al., 2013).

### 4.4 C‐S‐R strategies

We hypothesized in line with former findings, that the community‐level competitiveness and stress‐tolerance increases and the ruderality decreases during the old‐field succession (see Caccianiga, Luzzaro, Pierce, Ceriani, & Cerabolini, 2006; Navas et al., 2010; Prach & Pyšek, 1999; Prévosto et al., 2011). In contrast to this we found that the community‐level competitiveness remained stable and high during the succession when the alfalfa was included in analysis. The alfalfa is characterized with a pronounced C character, and was gradually replaced by perennial grasses with quite similar competitive abilities during the succession. She et al. (2009) found similar trends as the native perennial grass (*Stipa bungeana*) became dominant from the 10th year of succession in alfalfa fields. In our study, the subordinate species in young alfalfa fields were characterized by low competitive ability, while the perennial grasses, which increased their abundances parallel to the decline of alfalfa, had good competitive ability. This phenomenon is likely responsible for the increased C coordinates during the succession calculated without alfalfa.

In line with former studies, stress‐tolerant character of communities increased while the ruderal characters decreased with increasing field age (see Caccianiga et al., 2006; Navas et al., 2010; Prévosto et al., 2011) in analyses with and without alfalfa. Before establishing alfalfa fields, a soil disturbance by ploughing is typical; thus, R‐strategists can occur as first colonizers in young alfalfa fields raised from their generally persistent seed banks (Thompson, Bakker, Bekker, & Hodgson, 1998). Ruderals require regular soil disturbance, without this their gradual decline is typical (Deák et al., 2015). In parallel, stress‐tolerators can arrive from the vicinity by wind or by grazing animals and they can cope with the competition of grasses in the studied dry habitats; thus, their abundance increases during the succession (Dölle et al., 2008; Kelemen, Török, Valkó, Miglécz, & Tóthmérész, 2013). In our study, the replacement of alfalfa with native competitors and the increased cover of stress‐tolerators suggest that sowing of perennial legumes can accelerate grassland succession in this type of old‐fields (Li et al., 2008; Török et al., 2011; Van der Putten et al., 2000).

## 5 Conclusions

In this paper we studied the validity of widely accepted theories of classical old‐field succession studying perennial‐crop‐mediated succession using a functional approach including trait‐based functional changes and plant strategies. There are markedly different starting conditions in case of perennial‐crop‐mediated succession and classical old‐field succession where the initial vegetation is generally dominated by short‐lived species. In spite of these marked differences, we found striking similarities with classical old‐field successions in case of the changes of functional diversity, resource acquisition versus conservation trade‐off and seed dispersal strategies. However, we demonstrated that in perennial‐crop mediated succession there are remarkable differences compared to classical old‐field succession: We found that the competitive character of communities remained unchanged during the succession. This is well supported by the fact that the vegetation during the entire perennial‐crop‐mediated succession is characterized by perennials. Therefore, the initial stages of perennial‐crop‐mediated succession were similar to the middle stages of classical old‐field succession, with the marked difference that the vegetation changes could be much faster. The gradual colonization of native competitor grasses and native stress‐tolerators was typical parallel to the decline of alfalfa and ruderals. The alfalfa, in contrast to perennial mid‐succession grasses is a short‐lived perennial characterized by a typical decline of its stands after 3–5 years subsequently to its establishment and therefore, does not arrest the succession in an unwanted stage (see also Török et al., 2011). In support of the former findings we found increasing functional richness and divergence, but also unchanged or decreasing functional evenness. These results point out that in spite of the increasing replacement of alfalfa by perennial grasses and the unchanged competitive character of the community, the establishment of species with various functional characteristics is not limited. Our trait‐based analyses pointed out that the study of perennial‐crop‐mediated succession helps to fine‐tune the existing theories of old‐field succession by explaining the functional and dynamical processes in succession.

## Conflict of interest

None declared.

## Author Contributions

AK, BD, OV, TM, and PT contributed to sampling design and data collection. AK, BT, OV, TM, BD, and PT helped in analyses and data acquisition. AK, BT, OV, TM, BD, and PT contributed to manuscript preparation.
